# Editorial: Advances and Refinements in the Development and Application of Threshold of Toxicological Concern

**DOI:** 10.3389/ftox.2022.882321

**Published:** 2022-04-28

**Authors:** Grace Patlewicz, Andrew Worth, Chihae Yang, Tingting Zhu

**Affiliations:** ^1^ Independent Researcher, Durham, NC, United States; ^2^ European Commission, Joint Research Centre (JRC), Ispra, Italy; ^3^ Independent Researcher, Nürnberg, Germany; ^4^ The Procter & Gamble Company, Beijing, China

**Keywords:** TTC, Cramer classification, Toxtree, ecoTTC, internal TTC

The Threshold of Toxicological Concern (TTC) is an exposure threshold below which there is no appreciable risk to human health. There are two main approaches: TTC values based on cancer potency data from which a one in a million excess lifetime risk is estimated and TTC values based on non-cancer effects. For the latter approach, a distribution is typically fitted to the No Observed Adverse Effect Levels (NOAELs) from repeat dose toxicity studies from which a 5th percentile value is taken and adjusted using an uncertainty factor (usually this is 100). Established TTC values are those based on oral chronic studies that were first developed by Munro et al. (1996) who subcategorised chemicals into one of three Cramer structural classes (Cramer et al., 1978). Kroes et al. (2004) presented a tiered TTC approach that established several human exposure thresholds spanning four orders of magnitude where the lowest TTC was for substances presenting structural alerts for genotoxicity (0.15 μg/d), to the next tier for organophosphates/carbamates (18 μg/d) and the remaining higher TTC values representing the same three Cramer classes originally derived by Munro et al. (1996). The World Health Organization (WHO) and the European Food Safety Authority (EFSA) (European Food Safety Authority and World Health Organization, 2016; EFSA et al., 2019) have determined that the TTC approach is a sound and fit-for-purpose risk assessment tool, with a number of caveats, in cases where chemical-specific repeat dose toxicity data are not available.

More recent work has explored extending the dataset underpinning the original Cramer TTC values to demonstrate its protection and robustness (Yang et al., 2017). The work by Yang et al. (2017) was undertaken under the auspices of the EU SEURAT-1 project COSMOS whereupon a COSMOS TTC dataset was developed. This comprised 552 substances for which the toxicity data and chemical structural identifiers were carefully curated. This federated dataset was then used to derive new TTC values for two of the Cramer classes (I and III). The derived values (46 and 2.3 μg/kg bw/d respectively) have since been adopted as the current recommendations by the SCCS in relation to cosmetic substances (SCCS, 2021). In addition, a total of 476 chemicals from the RIFM (Research Institute for Fragrance Materials) dataset were added to the COSMOS/Munro TTC database for analysis, in particular bolstering Cramer class II chemicals from 40 chemicals to 111 chemicals. The derived TTC values of 49, 12.7, and 2.9 μg/kg bw/day for Cramer classes I, II or III confirm the adequacy of Munro TTC values and further support the use of TTC for safety assessment of fragrance materials (Patel et al., 2020). Thus, these above results show that cosmetic ingredients are sufficiently represented structural classes in the current TTC dataset that had been originally developed for food. The TTC concept is scientific and convincing for it to be applied in cosmetic safety assessment. In 2021, following the TTC updates in the 11th Notes of Guidance (SCCS, 2021), the TTC approach use in cosmetic safety assessment was adopted by the Technical Guidelines for Cosmetic Safety Assessment in China NMPA (National Medical Products Administration, 2021). It is anticipated that TTC relevant research work will be receive more attention in China going forward.

WHO/EFSA (European Food Safety Authority and World Health Organization, 2016) recommended that in the case of mixtures that are not fully defined, the evaluation should be considered on a case-by-case basis. In general, botanicals are complex mixtures with unknown constituents and lack of toxicity data. TTC expansion to botanicals and simple extracts became beneficial in the trend of cosmetic development and consumers’ need. Several research projects have been performed to establish safety exposure levels for botanical extracts used in cosmetics (Sica et al., 2018; Kawamoto et al., 2019; Mahony et al., 2020). Case studies of TTC application in botanical safety assessment have been recently published (Bury et al., 2021).

Others have investigated the relevance of the underlying Munro dataset to other sectors. In Nelms et al. (2019), the interest was in applying the TTC as an approach to prioritise large numbers of chemicals, specifically those on the active TSCA non-confidential inventory. New TTC values were established using the EPA’s Toxicity Values database (ToxValDB) which were similar though not identical to the original Munro TTC values.

In the case of cancer TTC, the Carcinogenicity Potency Database (CPDB) first developed by Gold et al. (1984) along with the methodologies (TD50 etc.) were updated as part of a CEFIC funded project (Boobis et al., 2017). An expert workshop was convened in April 2021 and new thresholds are being revisited, the work by Batke et al. (as described below) is one such example.

A similar approach, called ecoTTC, has been proposed for the establishment of environmental thresholds of concern (CITE). These can be derived for different species and modes of action. The underlying toxicity distributions can also be used to inform cross-species extrapolation. To date, the ecoTTC approach has not been used for regulatory purposes.

This research topic aimed to summarise a few of the recent developments in the area of TTC such as:

1) Addressing other routes of entry or exposure durations, e.g., TTCs for inhalation, internal vs. external TTCs.
2) Development and acceptance of TTC within different sectors, e.g., food safety, cosmetics, industrial chemicals.
3) TTC approach extensions to new chemical domains, e.g., organosilicon compounds (Schmitt et al., 2021). The organosilicon substances were analysed and demonstrated that Cramer Class III TTC would be protective for organosilicon chemistry.
4) Status on the development and application of the ecoTTC approach in environmental risk assessment.

Whilst TTC values for oral routes of entry are well established and have been accepted for specific risk assessment purposes, the same is not true for other routes of exposure. There have been efforts to derive TTC values for inhalation as a route of exposure but to date there is no consensus for what the harmonised values should be or whether applying the same means of subcategorising substances into Cramer structural classes is meaningful. In Nelms and Patlewicz, an attempt was made to derive new TTC values for the inhalation route and compare and contrast these against earlier attempts published previously by both Carthew et al. (2009) and Escher et al. (2010). The efforts highlighted a number of needs such as the role that study quality and inclusion/exclusion criteria plays in driving the conservatism of the resulting TTC values as well as alternative means to subcategorise substances on the basis of their structural characteristics. The preliminary study by Nelms and Patlewicz did at least foster discussion within the broader TTC community culminating in a new initiative to coordinate amongst various stakeholders including Cosmetics Europe and RIFM to compile and curate a federated dataset specific to inhalation repeat dose toxicity studies.

A natural evolution of the TTC concept is also to consider internal exposures. Instead of using chemical-specific external NOAELs (in µg/kg/day) in the TTC database, each of these values could be converted to plasma concentrations using either empirical *in vitro* data or estimated *in silico* values in conjunction with Physiologically based PharmacoKinetic (PBPK) models. Such an approach would also open the door for using other new approach methods in lieu of traditional toxicity data to derive TTC values or TTC-like values that could be useful for prioritisation as well as more nuanced risk assessment applications for cosmetics. In their article, Ellison et al. presented the state of the art for developing internal TTCs, the proof of concept study that they had been performing and how new *in vitro* caco-2 and *in vitro* hepatic metabolism studies being generated could be utilised with PBPK models to make the necessary conversions.

Similar to the work by Yang et al. (2017) and Nelms et al. (2019), Yamada et al. developed and curated a new database of oral subacute/subchronic studies for industrial chemicals of interest to the Japanese Chemical Substances of Control Law. The motivation in Yamada et al. was twofold; to compare TTC values derived using this new database relative to the original Munro dataset and to provide a resource that could be potentially merged with other databases to extend the chemical breadth and diversity to support new TTC derivations. Indeed, the newly derived TTC values from this dataset for Cramer III were comparable to other datasets such as the Munro et al. (1996), RepDose (Tluczkiewicz et al., 2011), OpenFoodTox (Reilly et al., 2019) and COSMOS (Yang et al., 2017) whereas the Class I values were somewhat lower. Yamada et al. surmised that this was due to the prevalence of industrial substances that are likely to have a higher toxicity than those in these other datasets which were more enriched by substances from food safety and cosmetics sectors.

The origins of the TTC were founded in food safety assessment. Serafimova et al. provided a state-of-the-art review to summarise the application of TTC in food safety from an EU perspective highlighting the different food sector applications, their reconciliation with the legal requirements in each case and what some of the future opportunities for TTC might be. The application of TTC spans more than just food contact or food flavouring ingredients—but also considers degradation products in plant protection products, flavourings in animal foodstuff as well as contaminants in food. In terms of future outlook, one of the key considerations they highlighted was to more accurately characterising relevant exposures. Various approaches could be taken here whether that be from the perspective of using an internal TTC approach such as that proposed by Ellison et al. or characterising the combined exposure to multiple chemicals with similar toxicological impacts.

Batke et al. performed an investigation of the applicability of using Cramer classes specifically for substances that were known non-genotoxic carcinogens. Following a detailed consistency check, a dataset of 137 non-genotoxic substances were collated. For these substances, a comparative analysis was performed between NOEL values, calculated effective tumor dose (ETD10) and benchmark dose levels derived by model averaging (BMDL10). There were 25 substances that had high bioaccumulation potential that occurred in the region of the 5th percentiles of the respective distributions. If these were excluded, then the 5th percentiles of the NOEL and BMDL10 values were comparable whereas the EDT10 was slightly higher but not statistically significantly so. Comparison of these distributions and their associated 5th percentiles with those of the original Cramer classes, notably Cramer class III since the majority of the substances from this dataset belonged to Cramer class III found that the TTC values were very similar. Although the TTC values were not the same, the range of TTC values expected following a random 5% removal showed that both datasets resulted in comparable values. This supported the hypothesis that the Cramer classes were sufficiently protective for non-genotoxic carcinogens.

The final article in the research topic was from Barron et al. who reviewed the derivation of ecoTTC values, providing an overview of the ecoTTC workflow, how values can be derived and what ongoing research is being undertaken.

In summary, this research topic provided some contrasting perspectives of the challenges in deriving TTCs, their applications and evolution to meet different use cases within human health and environment risk assessments. Clearly TTC has proven to be a useful tool in the risk assessment of chemicals in certain situations and continues to evolve as new toxicity data are acquired.
